# Bevacizumab treatment for advanced non-small cell lung cancer: A case report

**DOI:** 10.3892/ol.2013.1603

**Published:** 2013-10-04

**Authors:** YUN FAN, ZHIYU HUANG, WEIMIN MAO

**Affiliations:** Department of Chemotherapy, Zhejiang Cancer Hospital, Hangzhou, Zhejiang 310022, P.R. China

**Keywords:** anti-vascular endothelial growth factor, stable disease, safety of Avastin in lung trial, metastasis, chemotherapy

## Abstract

The safety of Avastin in lung cancer (SAiL) study is a multi-center, open-source, stand-alone study. Patients with untreated, locally advanced, metastatic or recurrent non-squamous non-small cell lung cancer (NSCLC) were administered up to six cycles of chemotherapy combined with bevacizumab-humanized monoclonal antibodies, followed by maintenance therapy with bevacizumab until further progression of the disease. From August, 2006 to July, 2008 there were a total of 2,172 patients enrolled in the study, with a median progression-free survival time of 7.8 months and an overall survival time of 14.6 months. The present study describes the case of a 54-year-old male with lung cancer and T3N0M1 subcutaneous metastasis, which was initially treated with bevacizumab-combined carboplatin/paclitaxel (C/P) therapy and then maintained solely with bevacizumab for five years. Following six cycles of C/P bevacizumab treatment, the therapeutic evaluation revealed a stable disease (SD). The patient was kept on bevacizumab maintenance therapy for 50 months without disease progression until a persistent 3+ proteinuria was diagnosed in a follow-up review, which led to bevacizumab withdrawal and concomitant tumor growth. The present study concluded that the long-term application of bevacizumab monoclonal antibodies (mABs) was safe in a late-stage non-small cell lung cancer patient. The major adverse reaction that was exhibited was proteinuria, which was associated with the cumulative dose of bevacizumab and was able to be reversed by withdrawal. Patients with a prolonged SD may benefit from bevacizumab maintenance therapy.

## Introduction

Among a variety of targeted anti-angiogenic drugs, the angiogenesis inhibitor, bevacizumab (Avastin), is the first to be confirmed to lead to a survival advantage in patients with advanced non-small cell lung cancer (NSCLC). The combination of chemotherapy and bevacizumab has become the new choice for advanced non-squamous NSCLC treatments (1). The safety of Avastin in lung cancer (SAiL) study (2) is a multi-center, open-source, stand-alone study. Patients with untreated, locally advanced, metastatic or recurrent non-squamous NSCLC were administered up to six cycles of chemotherapy combined with humanized monoclonal vascular endothelial growth factor A (VEGF-A) antibody (bevacizumab/Avastin) administration, followed by bevacizumab maintenance therapy until further progression of the disease. From August, 2006 to July, 2008, there were a total of 2,172 patients enrolled in the study, with a median progression-free survival (PFS) time of 7.8 months and an overall survival (OS) time of 14.6 months. The present study describes the case of one specific patient who participated in the SAiL research program. The patient presented with right lung adenocarcinoma and subcutaneous metastasis and had a PFS time of nearly five years while undergoing bevacizumab maintenance therapy following chemotherapy combined with bevacizumab.

## Case report

### Patient

**Figure 1:**
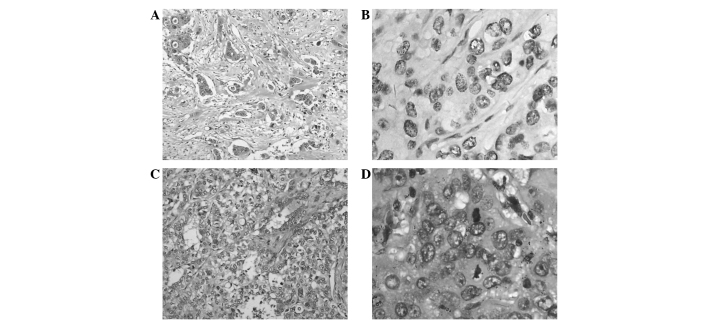
Bright field pathology images. (A) Primary lung tumor (HE staining; magnification, ×100). Tumor solid growth with evident differentiation. (B) Metastasis (IHC staining with TTF1-antibody showing positive tumor with high magnification (x400). The tumor demonstrated an increased size with evident differentiation. Mitosis was observed. (C) Metastasis (HE staining; magnification, ×100). Invasive growth of tumor cells in fibrous tissue. (D) Metastasis (HE staining; magnification, ×400). Tumor cells with an abundance of cytoplasm. Mitosis were observed. HE, hematoxylin and eosin; IHC, immunohistochemistry.

**Figure 2:**
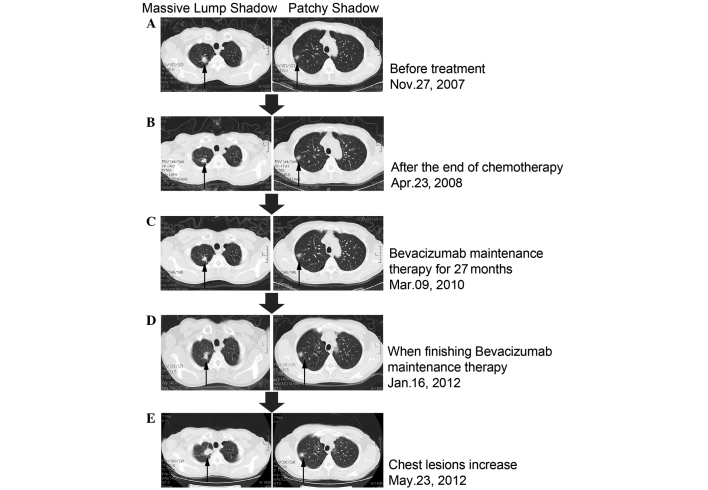
Chest computed tomography (CT) images of lung tumors at various stages of the treatment. (A) Pulmonary masses; (B) pulmonary masses slightly shrunk; (C) pulmonary masses stable; (D) pulmonary masses stable; (E) pulmonary masses clearly increased.

A 54-year-old male patient was admitted to the Zhejiang Cancer Hospital on October 16, 2007, due to a phyma at the left frontal brow. The phyma was 1.5 × 1.0 cm in size, with mild tenderness and no local redness. The patient presented with no abnormal coughing, sputum or fever. A surgical resection was performed on October 26, 2007, and the phyma was identified as a left frontal metastatic poorly-differentiated adenocarcinoma by pathology (Fig. 1A and B). Further examinations were performed in order to identify the primary tumor, and a chest computed tomography (CT) scan disclosed the presence of two lesions. One was a lump shadow with a small burr of ~2.0 cm in size in the upper right lung, while the other was also in the upper right lung, but was a patchy shadow with an unclear edge, thereby indicating a high possibility of lung cancer (Fig. 2A). Using emission computed tomography (ECT), several lightly visible restricted radioactivity concentration sites were detected at the left temporal bone, the 7th and 10th thoracic vertebra and the right ileum, while no evident abnormalities were observed in the brain. There were also no evident abnormalities in the pancreas, spleen, kidney or double-adrenal gland, which were examined by an ultra-B scan. The gastroscopic results and blood carcinoembryonic antigen (CEA) levels were normal and the patient had no history of smoking or high blood pressure.

### Physical examination

The general health of the patient was fine and the performance status (PS) was scored as 0. A surgical scar was observed on the left frontal area of the face. There was no enlargement of the two supraclavicular lymph nodes and heart and lung auscultation heralded negative results. No abnormality was observed by abdominal examination, routine blood tests, blood biochemical examination or routine urine tests. The patient was diagnosed with lung cancer combined with subcutaneous T3N0M1 metastasis.

### Treatment procedure

The patient was selected for the SAiL clinical trial. According to the SAiL scheme, the patient was initially administered 15 mg/kg (900 mg)d1 bevacizumab monoclonal antibody (mAb), 175 mg/m^2^ (300 mg)d1 paclitaxel and the area under the concentration time curve 6.0 (770 mg)d1 carboplatin.

Grade I bone marrow suppression and peripheral nerve toxicity and grade III hair loss and epistaxis developed following the chemotherapy. Following two chemotherapy cycles, the tumor in the upper right lung was observed to be slightly shrunken by chest CT, with the therapeutic evaluation of a stable disease (SD; Fig. 2). On January 11, February 1, February 28 and March 25, four chemotherapy cycles were applied as planned, resulting in six cycles in total. Compared with the pre-treatment evaluation, the therapeutic evaluation was of SD (Fig. 2B). On April 23, 2008, the patient was initially administered 15 mg/kg (900 mg/d1) bevacizumab mAb and then maintenance treatment every 3 weeks (Fig. 2B). The final bevacizumab mAb treatment was administered on January 16, 2012. The total bevacizumab mAb treatment time lasted 50 months, with a cumulative dose of 54 g. During the bevacizumab maintenance treatment, the patient was examined by chest CT every three months and by brain magnetic resonance imaging (MRI) every six months for the therapeutic evaluation. On January 16, 2012, the efficacy evaluation review using CT scans revealed the stable status of the tumor (Fig. 2D). The patient required to be withdrawn from the SAiL clinical trial due to long-lasting 3+ proteinuria. On May 23, 2012, a chest CT indicated that the upper right lung lesions were significantly increased compared with the previous data (Fig. 2E). The patient did not cough blood and had no fever, sputum, chest pain, hemoptysis, chest tightness, shortness of breath or headache. No abnormalities were identified from the brain MRI, bone ECT or abdominal CT, and the blood CEA was 33.2 ng/ml. A lung tumor biopsy was performed and cancer cells were identified. Considering the progress of the disease, the PFS was 54 months. On May 31, 2012, one chemotherapy cycle of gemcitabine (1250 mg/m^2^) combined with cisplatin (75 mg/m^2^) was administered and the patient developed grade III neutropenia and grade IV platelet decline. On July 17, 2012, a palliative treatment was performed on the right-sided lung cancer. Pathological lung nodules with poorly-differentiated adenocarcinoma cells combined with disintegration and necrosis were discovered forming two tumors, one small, sized 2 × 2 × 1.5 cm, and one large, sized 3.5 × 3 × 2.8 cm. Tumor invasion of the visceral pleura, infiltration and metastasis statuses were: bronchial roots lymph node 1/6, intrapulmonary bronchial lymph node 1/2, the 7th groups of lymph nodes 0/2, the 9th groups of lymph nodes 0/1 and the 10th lymph nodes 1/4 (Fig. 1C and D). G/C chemotherapies were performed on August 15, and September 6, 2012. The current Eastern Cooperative Oncology Group (ECOG) PS score was 1. Approval for the study was obtained from the ethical committee of Zhejiang Cancer Hospital and informed consent was obtained from all the participants.

### Adverse reactions to bevacizumab

**Figure 3:**
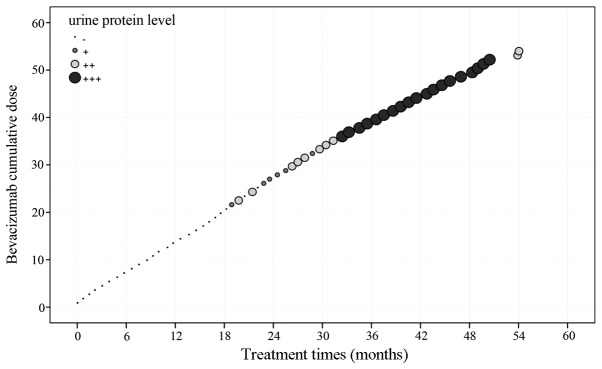
Urinary protein level positively correlated with the cumulative dose (grams) of bevacizumab (r=0.899; P=0.000).

During the bevacizumab treatment there was no sign of hemoptysis. During the 1–2 cycles of chemotherapy, grade I epistaxis was observed, but no high blood pressure. During cycles 1–17 of bevacizumab treatment, there was no significant proteinuria. Weak positive proteinuria was diagnosed from the start of the 18th cycle, but from cycles 33–39, the proteinuria was 1.0–2.0 g/l (++). Following several 24-h interval examinations, the total urine protein was shown to peak at 1.38 g/24 h. During cycles 40–57, the proteinuria was 2.0–4.0 g/l (+++). The total urine protein measurement at the 57th cycle was 2.284g/24 h on January 17, 2012. The urinary protein level in the patient demonstrated a positive correlation with the cumulative dose of bevacizumab (P=0.000; Fig. 3). Subsequent to withdrawing the medication and pausing for more than a month, proteinuria was diagnosed [<0.15 g/l (−)] on May 25, 2012, and was kept at a constant negative from then on. In this patient with advanced non-small cell adenocarcinoma and a PS score of 0, bevacizumab combined with paclitaxel and carboplatin was able to stabilize the tumor condition. The bevacizumab maintenance therapy lasted for 50 months. At three months after treatment withdrawal the tumor progression restarted. Overall, the bevacizumab therapy demonstrated a good long-term effect with the major adverse reaction of proteinuria correlating with the drug cumulative dose, which may be completely restored following withdrawal.

## Discussion

There are several studies with regard to bevacizumab therapy. Sandler *et al* reported that when comparing bevacizumab-based monoclonal antibody therapies combined with C/P with solely C/P chemotherapies, the former was able to significantly increase the objective response rate (35% vs. 15%; P<0.0001) and PFS time (6.2 months vs. 4.5 months; P<0.001). The treatment may also significantly increase the survival length, with a mean of 12.3 months compared with 10.3 months (P=0.001) (3,4). The present study is the first advanced NSCLC treatment study, which extended the survival time to more than a year. Based on the results of phase III clinical studies, the U.S. Food and Drug Administration (FDA) has approved the combination of bevacizumab with C/P as the first-line therapy for advanced non-squamous non-bleeding NSCLC (3). In 2007, in a European multi-center based phase III clinical trial, Reck *et al*(5,6) reported the survival differences between two doses of bevacizumab and gemcitabine/carboplatin (G/C) treatments (7.5 mg/kg and 15 mg/kg) and a pure G/C chemotherapy. This pilot study was performed using 1,043 cases of previously untreated advanced non-squamous NSCLC without brain metastases. The results demonstrated that chemotherapy combined with various doses of bevacizumab may significantly prolong disease PFS. The low-dose group had a 25% lower risk of mortality (HR=0.75; P=0.003) and a PFS time of 6.7 months compared with 6.1 months in the placebo group, while the high-dose group with a PFS time of 6.5 months had an 18% lower risk of mortality compared with the placebo group (HR=0.82; P=0.83). Currently, pre-clinical studies have confirmed that subsequent to stopping an anti-VEGF treatment, the tumor blood vessels tend to re-grow and the structures of re-grown tumor vascularization are similar to patients without the anti tumor vascularization treatment, while the VEGF-A expression and VEGF dependence are also noticed (7–10). These findings provide the theoretical rationale of a bevacizumab maintenance therapy and explain why bevacizumab may extend the disease PFS time. In 2011, Nadler *et al* reported in the results from the U.S. community practice network that advanced NSCLC may achieve a favorable clinical outcome following the completion of chemotherapy with bevacizumab maintenance therapy until further disease progression (11). Almost all clinical studies have used bevacizumab combined with first-line chemotherapies following maintenance therapy, hence the National Comprehensive Cancer Network (NCCN) and European Society for Medical Oncology (ESMO) guidelines and others recommend the use of bevacizumab maintenance therapy once the disease is stabilized by a first-line chemo-treatment. However, a clear long-term beneficial effect of bevacizumab maintenance therapy has not yet been confirmed. The present case was part of the SAiL (2) clinical trial, and the primary endpoint results suggested that bevacizumab combined with chemotherapy regimens may delay the time to progression (TTP) by 7.2–7.8 months and achieve 14.6–15.3 months OS. The objective response rates (ORR) and disease control rates (DCR) were as high as 50.8 and 88.7%, respectively. These data revealed a breakthrough compared with previous chemotherapy effectiveness levels (ORR, 30–35%; TTP or PFS, 4–6 months; and OS, 9–11 months) (12–17).

Following a C/P-combined bevacizumab treatment, the tumor status of the patient in the present study was stable. The bevacizumab maintenance therapy lasted for the long duration of 50 months, and three months subsequent to withdrawing the treatment due to proteinuria, the disease re-started progression. The total PFS was 54 months, which was much longer than the reported levels and confirms the safeness of long-term bevacizumab application. Although the tumor did not significantly narrow, it was maintained in a long progression-free period and the results agree with the ‘survival with tumor’ concept. This concept refers to the survival of advanced patients through effective anti-tumor treatment to prolong the stable disease. The tumor re-start progress following the withdrawal of bevacizumab also illustrates that the prolonged SD status is associated with bevacizumab maintenance therapy. The present case revealed that the proteinuria level was positively correlated with the cumulative bevacizumab dose and therefore may contribute to side-effect studies of bevacizumab in the future.

In summary, bevacizumab maintenance therapy was identified to be correlated with a 54-month prolonged PFS in the patient of the present study. The long-term use of bevacizumab led to a certain level of adverse reaction, which was controlled and completely reversed subsequent to withdrawing the treatment. Currently there is no clear indication for using bevacizumab in NSCLC treatments and therefore the present case of prolonged PFS may only be an individual case. A large-scale clinical trial is necessary to validate the possibility of using bevacizumab as a standard maintenance therapy for lung cancer.

## References

[1] Bareschino MA, Schettino C, Rossi A (2011). Treatment of advanced non small cell lung cancer. J Thorac Dis.

[2] Crinò L, Dansin E, Garrido P (2010). Safety and efficacy of first-line bevacizumab-based therapy in advanced non-squamous non-small-cell lung cancer (SAiL, MO19390): a phase 4 study. Lancet Oncol.

[3] Sandler A, Gray R, Perry MC (2006). Paclitaxel-carboplatin alone or with bevacizumab for non-small-cell lung cancer. N Engl J Med.

[4] Sandler A, Yi J, Dahlberg S (2010). Treatment outcomes by tumor histology in Eastern Cooperative Group Study E4599 of bevacizumab with paclitaxel/carboplatin for advanced non-small cell lung cancer. J Thorac Oncol.

[5] Reck M, von Pawel J, Zatloukal P (2009). Phase III trial of cisplatin plus gemcitabine with either placebo or bevacizumab as first-line therapy for nonsquamous non-small-cell lung cancer: AVAil. J Clin Oncol.

[6] Reck M, von Pawel J, Zatloukal P, BO17704 Study Group (2010). Overall survival with cisplatin-gemcitabine and bevacizumab or placebo as first-line therapy for nonsquamous non-small-cell lung cancer: results from a randomised phase III trial (AVAiL). Ann Oncol.

[7] Yuan F, Chen Y, Dellian M (1996). Time-dependent vascular regression and permeability changes in established human tumor xenografts induced by an anti-vascular endothelial growth factor/vascular permeability factor antibody. Proc Natl Acad Sci USA.

[8] Mancuso MR, Davis R, Norberg SM (2006). Rapid vascular regrowth in tumors after reversal of VEGF inhibition. J Clin Invest.

[9] Bagri A, Berry L, Gunter B (2010). Effects of anti-VEGF treatment duration on tumor growth, tumor regrowth, and treatment efficacy. Clin Cancer Res.

[10] Ebos JM, Lee CR, Cruz-Munoz W (2009). Accelerated metastasis after short-term treatment with a potent inhibitor of tumor angiogenesis. Cancer Cell.

[11] Nadler E, Yu E, Ravelo A, Sing A, Forsyth M, Gruschkus S (2011). Bevacizumab treatment to progression after chemotherapy: outcomes from a U.S. community practice network. Oncologist.

[12] Kelly K, Crowley J, Bunn PA (2001). Randomized phase III trial of paclitaxel plus carboplatin versus vinorelbine plus cisplatin in the treatment of patients with advanced non - small-cell lung cancer: a Southwest Oncology Group trial. J Clin Oncol.

[13] Scagliotti GV, De Marinis F, Rinaldi M, Italian Lung Cancer Project (2002). Phase III randomized trial comparing three platinum-based doublets in advanced non-small-cell lung cancer. J Clin Oncol.

[14] Schiller JH, Harrington D, Belani CP, Eastern Cooperative Oncology Group (2002). Comparison of four chemotherapy regimens for advanced non-small-cell lung cancer. N Engl J Med.

[15] Fossella F, Pereira JR, von Pawel J (2003). Randomized, multinational, phase III study of docetaxel plus platinum combinations versus vinorelbine plus cisplatin for advanced non-small-cell lung cancer: the TAX 326 study group. J Clin Oncol.

[16] Gridelli C, Ardizzoni A, Douillard JY (2010). Recent issues in first-line treatment of advanced non-small-cell lung cancer: Results of an International Expert Panel Meeting of the Italian Association of Thoracic Oncology. Lung Cancer.

[17] Scagliotti GV, Parikh P, von Pawel J (2008). Phase III study comparing cisplatin plus gemcitabine with cisplatin plus pemetrexed in chemotherapy-naive patients with advanced-stage non-small-cell lung cancer. J Clin Oncol.

